# Plasma cholinesterase activity as diagnostic marker for systemic inflammation

**DOI:** 10.1186/cc13409

**Published:** 2014-03-17

**Authors:** AR Zivkovic, K Schmidt, T Brenner, S Hofer

**Affiliations:** 1 Universitätsklinikum Heidelberg, Germany

## Introduction

Systemic inflammation is a generalized response to internal or external inflammatory stimuli often resulting in multiple organ failure. Therefore, early diagnosis of systemic inflammation would be of great therapeutic and prognostic importance. Various inflammation biomarkers have been used in clinical and experimental practice, but a definitive diagnostic tool for an early detection of systemic inflammation remains to be identified. The neurotransmitter acetylcholine (Ach) has been shown to play an important role in the inflammatory response. Serum cholinesterase (butyrylcholinesterase (BChE)) is synthesized in the liver and acts as the major Ach hydrolyzing enzyme in plasma. Hence, BChE activity has been widely used as a biomarker for liver function. However, the role of this enzyme in the inflammatory pathomechanism has not yet been fully understood. Here, we describe a strong correlation between the BChE activity and the systemic inflammatory response.

## Methods

In this study we measured BChE activity in healthy subjects and in critically ill patients, clinically diagnosed with systemic inflammation or sepsis. Furthermore, we measured the levels of routine inflammation biomarkers and liver function parameters in blood of critically ill patients. Data were statistically analyzed using unpaired Student t test, Pearson correlationor ANOVA followed by Dunnett's post hoc analysis. *P *< 0.05 was considered statistically significant.

## Results

We found that the activity of BChE in patients with systemic inflammation was dramatically reduced in comparison with healthy subjects and negatively correlated with the serum levels of conventional inflammatory biomarkers. BChE synthesis depends on hepatic function. Surprisingly, the observed reduction in the BChE activity during systemic inflammation does not correlate with liver failure.

## Conclusion

Our results suggest that BchE activity might play an important role in the diagnosis of systemic inflammation independent of overall hepatic function.

